# After the blades: The late MIS3 flake-based technology at Shuidonggou Locality 2, North China

**DOI:** 10.1371/journal.pone.0274777

**Published:** 2022-10-12

**Authors:** Peiqi Zhang, Nicolas Zwyns, Fei Peng, Sam C. Lin, Corey L. Johnson, Jialong Guo, Huiming Wang, Xing Gao

**Affiliations:** 1 Department of Anthropology, University of California, Davis, Davis, California, United States of America; 2 Key Laboratory of Vertebrate Evolution and Human Origins of Chinese Academy of Sciences, Institute of Vertebrate Paleontology and Paleoanthropology, Chinese Academy of Sciences, Beijing, China; 3 Department of Archaeology and Museology, School of Ethnology and Sociology, Minzu University of China, Beijing, China; 4 Centre for Archaeological Science, School of Earth, Atmospheric and Life Sciences, University of Wollongong, Wollongong, Australia; 5 Australian Research Council Centre of Excellence for Australian Biodiversity and Heritage, University of Wollongong, Wollongong, Australia; 6 Institute of Culture Relics and Archaeology of Ningxia Hui Autonomous Region, Yinchuan, China; 7 The University of Chinese Academy of Sciences, Beijing, China; New York State Museum, UNITED STATES

## Abstract

Contrasting with the predominance of blade-based assemblages in the Eurasian Upper Paleolithic, the large-scale persistence of a core-and-flake technology remains one of the defining features of Late Pleistocene lithic technology in East Asia. In North China, Shuidonggou is an exceptional site where both technologies are documented, therefore, it is an important archaeological sequence to understand regional technological evolution during the Marine Isotopic Stage 3. Blade technology first occurred at Shuidonggou Locality 1 and 2 around 41 ka cal BP while core-and-flake assemblages were widespread in North China. However, systematic technological studies on assemblages postdating 34 ka cal BP have not been conducted to examine whether the blade technology appeared and disappeared over a short yet abrupt episode, or persists and integrates into other forms in the region. Here, we conducted qualitative and quantitative analyses to reconstruct lithic productions on the assemblages at Shuidonggou Locality 2, dated after 34 ka cal BP. Our results show that there is a total absence of laminar elements in stone artifacts dated to 34–28 ka cal BP at Shuidonggou. Instead, we observe a dominance of an expedient production of flakes in the younger assemblages, illustrating a rapid return to flake-based technology after a relatively brief episode of stone blade production. Combining archaeological, environmental, and genetic evidence, we suggest that this technological ‘reversal’ from blades back to core and flake technology reflect population dynamics and adaptive strategies at an ecological interface between East Asian winter and summer monsoon.

## Introduction

In Paleolithic studies, the definition of periods or cultural units is generally based on the form and manufacturing techniques of objects made from stone and other materials, which change over time and space. In most regions of Eurasia, there is a three-periodization system with the Lower, Middle, and Upper that is characterized by different technological traditions. Yet in East Asia, the core-and-flake assemblages (CFAs), also known as the ‘small flake industry’ in North China, or the ‘large pebble tools’ in South China, are widespread and persistent from the Early Pleistocene to the Last Glacial Maximum or even the Holocene [1–6]. The informal and expedient character of the CFAs suggest a persistence of relatively simple stone tool production systems that stands in contrast with the diverse and widespread technological transitions of the Paleolithic record seen elsewhere in Eurasia amid discussions on regional variations [1, 7–9].

Why the CFAs persisted for such a long time in East Asia is still a matter of debate, with numerous hypothetical explanations yet to be formally tested. The lack of change in lithic technology may be related to low population densities that inhibited cultural innovation/transmission [10], or on the contrary, the maintenance of social norms among phylogenetically connected populations [2, 11, 12]. Other claims invoked technical constraints imposed by the quality of stone raw materials [13, 14]; the reliance on organic materials such as wood or bamboo [15–17]; similarities in mobility patterns [2]; adaptations to low environmental pressures and low-intensity resource exploitation [2, 18]. Vaquero and Romagnoli argued that there is unlikely a single explanation for the longstanding behavior of technological expediency [19], and a minimal investment strategy for stone tool production actually could be a baseline in human behavior. From this perspective, it may be worth investigating the conditions and constraints that gave rise to high-cost technologies or shifts between high- and low-cost systems. Hence, looking for evidence of such trade-offs is important to understand the nature of East Asian CFAs. Therefore, analyzing the CFAs occurred alongside other lithic technologies allow us to better evaluate factors that underlie the continuity and variations (or a lack thereof) of lithic technologies in East Asia.

**Fig 1:**
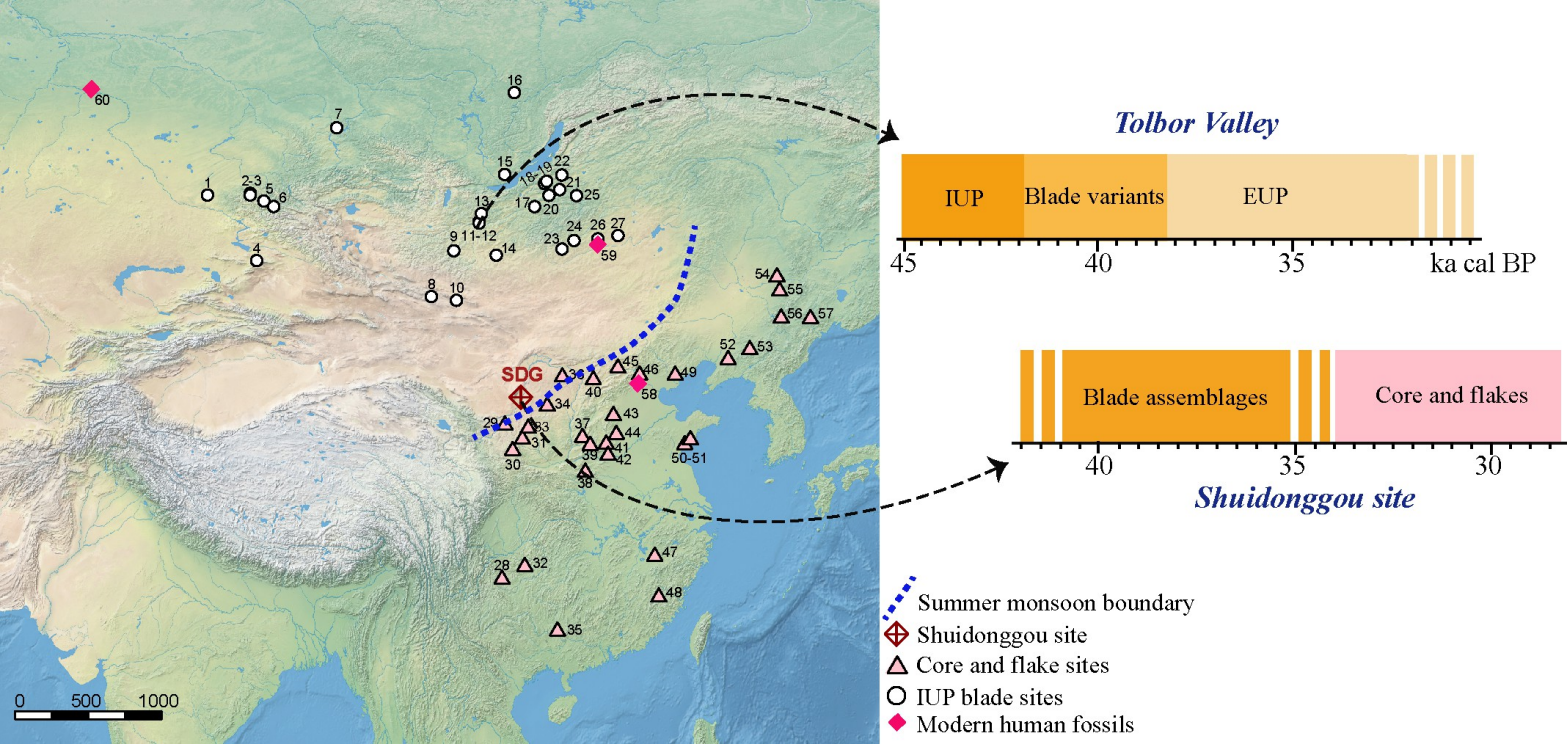
Distribution of MIS3 CFAs and IUP assemblages and the chrono-cultural sequence at Shuidonggou site and in the Tolbor Valley of North Mongolia. **IUP blade sites:** 1. Kara-Tenesh, 2. Denisova Cave, 3. Ust’Karakol, 4. Ushbulak, 5. Kara-Bom, 6. Malo Yaloman Cave, 7. Kurtak 4, 8. Chikhen 2, 9. Tsatsyn Ereg, 10. Tsagaan Agui, 11. Tolbor 21, 12. Tolbor 16 and 4, 13. Egiin-Gol (Dorolj 1–2), 14. Moyl’tynam, 15. Arembovski, 16. Makarovo 4, 17. Podzvonkaya, 18. Vavarina Gora, 19. Kamenka A, 20. Kandabaevo, 21. Tolbaga, 22. Khotyk, 23. Khanzat-1, 24. Rashaan Khad, 25. Barun-Alan, 26. Khavsgayt (and Salkhit), 27. Otson Tsokhio16–18; **Core and flake sites dated to 50–30 ka:** 28. Laoya Cave, 29. TX08&03, 30. Changweigou, 31. Xujiacheng, 32. Ma’anshan, 33. Liujiacha, 34. Salawusu, 35. Bailiandong Cave, 36. Wulanmulun, 37. Dingcun (7701), 38. Longquandong, 39. Fuyihe (Xiachuan), 40. Shiyu, 41. Zhiji Cave. 42. Zhaozhuang, 43. Dangcheng (Shidie), 44. Xiaonanhai, 45. Xibamaying, 46. Upper cave, 47. Xianrendong Cave, 48. Wanshouyan (Chuanfan Cave), 49. Zhuacun, 50. Huangniliang, 51. Dazhushan, 52. Xiaogushan, 53. Miaohoushan, 54. Yanjiagang, 55. Zhoujiayoufang, 56. Xianrendong, 57. Shimenshan. **Modern human fossils:** 58. Tianyuan Cave, 59. Salkhit, 60. Ust’-Ishim. Map: https://www.naturalearthdata.com.

One of the clear examples of technological changes among the CFAs in East Asia is the appearance of blade technology in North China during the late Marine Isotopic Stage 3 (MIS3). At the Shuidonggou (SDG) site complex, varying numbers of blade-related artifacts have been reported from three localities–SDG locality 1 (SDG1), SDG locality 2 (SDG2), and SDG locality 9 (SDG9). Apart from SDG, contemporaneous lithic assemblages in North China are generally characterized by a continuity of CFAs (Fig 1), lacking classic features and evidence of blade technology. In this context, the sudden appearance of blades in North China has been considered as a southward extension of the Initial Upper Paleolithic (IUP) coming from the north. In the Steppe belt (Fig 1), IUP blade assemblages are mostly dated between 48–40 ka cal BP [20–28]. At SDG, the blade technology may be as old as 41 ka cal BP [9, 28–32], however, it becomes elusive after 34 ka cal BP (Fig 1). Assemblages from SDG2 dated after 34 ka cal BP are typologically similar to the CFAs, and so are the younger CFAs at SDG8 [33].

Such a technological ‘reversal’ at SDG2 is against the expectations drawn from the general development of the Upper Paleolithic elsewhere in Eurasia, where a heavy reliance on laminar production is widely observed [34–36]. In the neighboring Steppe belt, from where blades in North China likely originate, blade production was persistent and gave way to the Early Upper Paleolithic varieties of blade and bladelet reduction systems [23, 37, 38]. In some regions, bladelet technology may have developed at the expense of larger blades but there is no doubt that both technologies coexisted during MIS3. In contrast in North China, the lithic sequence at SDG2 appears to reflect a peculiar case in which blade technology plays a rather marginal role. What remains unclear is whether the technology only appeared over a short period, or if it persisted in some form that has yet to be recognized. These answers have different, yet major implications for adaptive and population dynamic scenarios.

Clarifying the nature of the technological shift at SDG is therefore critical for understanding human cultural adaptations and population dispersals in the region. The millennial-scale climate fluctuations of the MIS3 may have affected the environmental settings around and beyond SDG [39, 40]. These uncertainties may have further forced changes in human movements and technological behaviors. More specifically, a geographic pattern of technological distribution emerged during the late MIS3 (Fig 1), between the East Asian winter monsoon region and summer monsoon region. In addition, ancient DNA studies have partly revealed how complex the population movements and interactions may have been in the region in the period of the MIS3. Between 40–30 ka cal BP, a modern human lineage represented by the fossil of Tianyuan Cave and AR33K in Amur region (Heilongjiang, China) rose in North China [41–43] and expanded toward East Mongolia, as shown by the genome of the Salkhit individual [44]. These changes in population are contemporaneous with the geographical clustering between the two technological systems, and the technological ‘reversal’ to CFAs that appears to take place at SDG2. The Tianyuan lineage is distinct from the populations associated with the IUP blade assemblages in the Steppe belt and dates to ca. 45 ka cal BP, based on the modern human remains from Ust’-Ishim, Siberia, or from Bacho Kiro Cave, in Eastern Europe [45–47].

Finding out whether there are any relics of blade technology in the CFAs at SDG2 would be informative for the technological evolution, the status of CFAs, and population dynamics in and beyond North China during late MIS3. What does such a technological shift mean in the broader scenario of cultural evolution and human behavior in and beyond North China? If the blade technology did continue to take hold, one set of possibilities is the lithic composition at SDG2 represents either a sampling bias due to site taphonomy, site function, and/or visibility and preservation that is devoid of blade products. Under these situations, however, we would still expect to find at least some technological elements associated with blade production, similar to other Upper Paleolithic assemblages elsewhere in Eurasia [35]. Otherwise, an absolute absence of any blade elements would indicate an abrupt technological discontinuity and replacement by the CFAs, which might be a response to environmental pressures, change and movement of human populations, or shifts in cultural innovation and convention associated with demographic structure, or a combination of the reasons above.

To evaluate these scenarios requires a better understanding of the technological variation of the CFAs at this site, beyond the general label of the simple CFA technology. At SDG2, the characterization of the CFAs is based on few studies [48, 49], and what happened to the blade technology after 34 ka cal BP remains unclear. Here, we conduct an in-depth analysis on the stone artifacts from the four upper layers excavated from SDG2 during 2014–2016 field seasons. First, we examine whether any technological features of blade technology leave impacts in the younger cultural layers. The second aim is to explore whether there are technological variations within the CFAs with respect to blank production, core reduction, tool modification, and raw material utilization. We further discuss the Upper Paleolithic technological variation at SDG2 under the context of environmental change and population dynamics during the late MIS3.

## Material and methods

### Material

**Fig 2:**
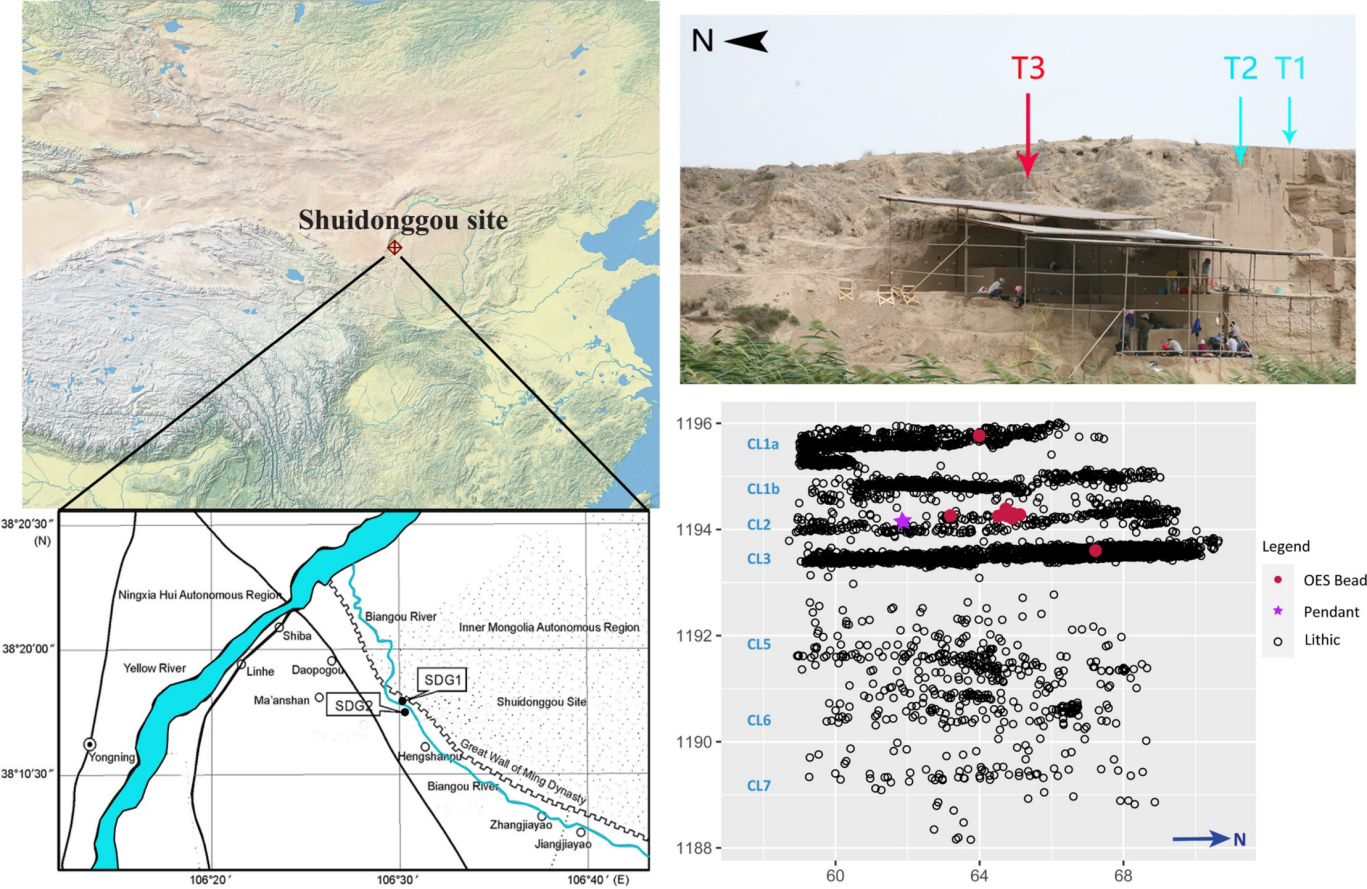
Location of Shuidonggou Locality 2 and cultural layers (adapted from [30, 50]).

SDG is a site complex with 12 localities on the eastern Loess Plateau, about 18 km east of the Yellow River in North China (Fig 2). Among the various localities that have been discovered, SDG1, SDG2, and SDG9 yielded varying numbers of blade artifacts. The Pleistocene assemblage at SDG1 is often assigned to be IUP based artifacts [20, 28, 51–53], while at SDG2, the relevant material is restricted to a few blade-related artifacts found in the two lower layers of CL5 and CL7 [9, 54]. SDG2 was excavated between 2003 and 2007 at Trench 1 and 2 and between 2014 and 2016 at Trench 3 (Fig 2). Based on the distribution of cultural remains and the sediment depositional sequence, seven cultural layers (CL) were identified at the site. CL7-CL1 (CL1 including two sub-layers CL1a and CL1b) are recognized across all three excavated trenches, while CL4 is only present in Trench 1 [9, 30]. The deposition of the site is associated with two general sedimentary processes: the two lower CLs 6–7 are composed of lacustrine sediments dated to ca. 41–34 ka cal BP; the upper layers (CL5-CL1a) were deposited in a loess-like matrix dated between 34–28 ka cal BP [30, 32]. Sedimentological and pollen analyses suggest that CL3 and CL2 accumulated under a relatively warm and humid condition with a forest-steppe vegetation, and likely deposited under a lakeshore context [51]; computer simulation of artifact deposition among CL3-CL1a show signs of a very low-energy water movement that only slightly changed artifact orientations [55]. The Loess Plateau shifted to a colder and drier environment around ca. 29 ka starting from MIS2 [51, 56].

Stone artifacts uncovered from the first excavation between 2003 and 2007 are mostly CFAs, with only a few blades and blade cores identified in CL5 and CL7 from Trench 2. While it has been reported that blade technology is absent in the upper layers at SDG2 [54], elongated artifacts that meet the metric definition of blades still occur, at least occasionally [57]. Hence, how long blade reduction systems subsist after their first introduction in the region remains unclear. Beside stone artifacts, SDG2 also yielded faunal remains, charcoals, ostrich eggshell fragments, beads made from ostrich eggshells and freshwater mollusks, and combustion features [54, 58, 59]. The beads are associated with the CFAs that post-date the IUP-like blade artifacts at Trench 2 of SDG2.

The material presented here was collected from Trench 3 at SDG2 during the 2014–2016 excavation program (Fig 2). Artifacts with a maximum dimension equal to or larger than 2 cm were piece-plotted by a total station, while objects smaller than 2cm were collected as aggregate samples in a screen, sorted by sedimentary layers and by arbitrary spits (5 cm intervals) within excavation units (1 m^2^). This excavation yielded numerous cultural remains, including lithics, faunal remains, charcoals, combustion features, ostrich eggshell fragments and beads, and a bone pendant; the latter two types of ornaments being found in the upper layers. In total, the Trench 3 excavation yielded 4784 piece-plotted stone artifacts. In this study, we focus on the 4327 lithics from the four upper layers (CL3-CL1a) (Table 1). The studied material is curated at the Institute of Vertebrate Paleontology and Paleoanthropology (IVPP), Chinese Academy of Sciences in Beijing, China. All necessary permits were obtained from the IVPP and the study complies with all relevant regulations. These stone artifacts have not been comprehensively studied for their technological characteristics. The investigation on the upper layers is informative about what happened after the appearance of the blade technology and technological variations of the early Upper Paleolithic, as well as the human response to environmental change during the late MIS3 in North China.

**Table 1:** The studied lithic sample of SDG2.

	CL1a		CL1b		CL2		CL3		Total	
	*N*	*f*	*N*	*f*	*N*	*f*	*N*	*f*	*N*	*f*
Core	40	8%	36	6%	46	8%	239	9%	361	8%
Flake	56	12%	338	53%	218	40%	887	33%	1499	35%
Tool	21	4%	24	4%	49	9%	198	8%	292	7%
Hammer	-	-	1	0.2%	-	-	9	0.4%	10	0.3%
Shatter	329	67%	217	34%	192	37%	1139	43%	1877	43%
Manuport	42	9%	21	3%	32	6%	193	7%	288	7%
Total	488		637		537		2665		4327	

### Methods

The artifacts were analyzed by recording standard attributes as defined in Inizan et al., (1999) [60], Andrefsky (2005) [61], using the E4-MSAccess database software (https://oldstoneage.com/). The data were then described at an assemblage level by their respective cultural layers (summarized in S1 Table). We summarized the attribute data of flakes and flake tools based on three categories which are indicated below: all artifacts including fragments, artifacts with striking platform preserved (also as the minimum number of identified flakes), and complete pieces. Descriptive statistics and statistical tests were applied to describe numerical variables and frequencies of categorical attributes at the assemblage level. We used the Mann Whitney U Test (Wilcoxon Rank Sum Test) to evaluate the difference of numerical measurements between/within the categories: paired test to evaluate measurements of individual blanks and cores; unpaired test for measurements between blanks and tools. One-way ANOVA was used to compare numerical data across multiple categories of core types. An alpha level of 0.05 was adopted for assigning statistical significance, though we further correct the critical threshold to 0.005 by the Bonferroni correction procedure to account for multiple testing (number of tests = 10) [62]. In addition to quantitative analyses, we also qualitatively describe the typology, technique, and production technology. Together, the quantitative and qualitative approaches provide a holistic reconstruction of the lithic reduction sequences (*Chaîne Opératoire*) across the studied cultural layers represented at the site. We also assessed the diachronic pattern of lithic productions and changes in technological behavior.

## Results

### The composition of lithic assemblage

**Fig 3:**
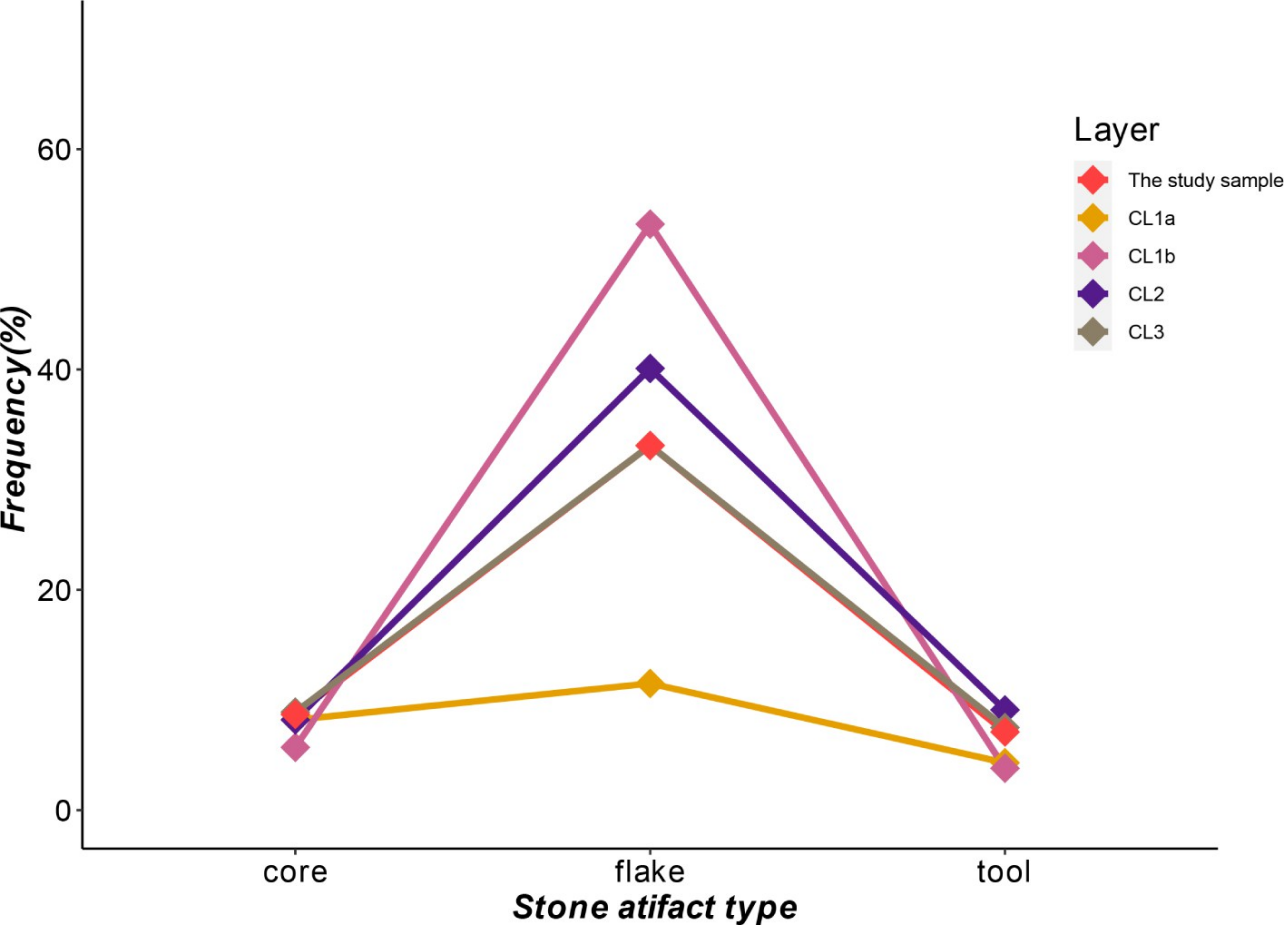
Frequencies of core, flake, and tool across layers.

The study sample consists of shatters and flakes/blanks, followed by cores, retouched tools, manuport, and hammerstones (Table 1). Major artifact types are blanks, cores, and tools. After calculating the frequency of each major artifact type relative to the assemblage from every layer, we observed an increase in flake frequency and a relatively small and gradual decrease in retouched tool frequency from CL3 to CL1b (Fig 3). In terms of core frequency, it is constant over the layers other than in CL1b, which has a lower frequency. CL1b in particular shows the lowest frequency of cores and tool modifications but the highest flake frequency indicating a pattern of high productivity in blank production with a low tool modification rate. On the other hand, CL1a shows low productivity in both blanks and tools. The profile of CL3 is nearly identical to the structure of the whole SDG2 assemblage, but the fact that the sample size of this CL is very close to the studied SDG2 assemblage here is probably responsible for the similar proportion structure.

In terms of raw material, siliceous limestone is the most represented material (N = 1651, 38.2%), followed by chert (N = 1104, 25.5%), quartzite (N = 909, 21%), sandstone (N = 509, 11.8%), and quartz (N = 67, 1.5%) from the local riverbed. These frequencies are in line with those described for the lithic assemblages recovered from Trench 1 and 2 in previous excavations [54, 63]. From CL3 to CL1a, we observe an increase in the use of quartzite and sandstone and a decrease in the use of siliceous limestone and chert. Retouched tools were mostly made in siliceous limestone and chert (S2 Table). In general, the use of raw material had few changes through time at SDG2. The frequency of archeological raw material differs to the observation from a recent raw material survey, which showed a dominance of siliceous limestone (49.5%) and quartzite (35.5%) but fewer sandstones (7.5%) and cherts (4%) on the landscape [64]. Non-local fine-grained chert artifacts were exclusively found in CL2 in a low frequency from our study sample and the previous excavation [63], most of which either are retouched or show signs of use [65].

### Blank production

#### Blank configuration and size

The upper layer assemblages contain 1499 blanks, around half of which are complete (N = 752, 40%) (Table 2). The other half of the blank assemblage is composed of fragments, including many split blanks (vertical breakages and Siret accidents).

**Table 2:** The frequency of blanks and tools.

		CL1a		CL1b		CL2		CL3		Total	
		*N*	*f*	*N*	*f*	*N*	*f*	*N*	*f*	*N*	*f*
Non-retouched blanks	Complete	25	45%	148	44%	115	53%	464	52%	752	50%
	Proximal	5	9%	34	10%	13	6%	75	8%	127	8%
	Complete split	12	21%	53	16%	24	11%	103	12%	192	13%
	Proximal split	-	-	7	2%	1	0.5%	22	2%	30	2%
	Distal	4	6%	41	12%	29	13%	92	10%	166	11%
	Undetermined	10	18%	65	19%	36	18%	131	15%	232	15%
	Total	56	100%	338	100%	218	100%	887	100%	1499	100%
Retouched tools	Complete	7	50%	8	40%	30	70%	72	51%	117	54%
	Proximal	1	7%	1	5%	2	5%	13	9%	17	8%
	Split	4	29%	6	30%	2	5%	15	11%	27	12%
	Distal	1	7%	-	-	5	11%	19	14%	25	12%
	Undetermined	1	7%	5	25%	4	9%	21	15%	31	14%
	Total	14	100%	20	100%	43	100%	140	100%	217	100%

**Fig 4:**
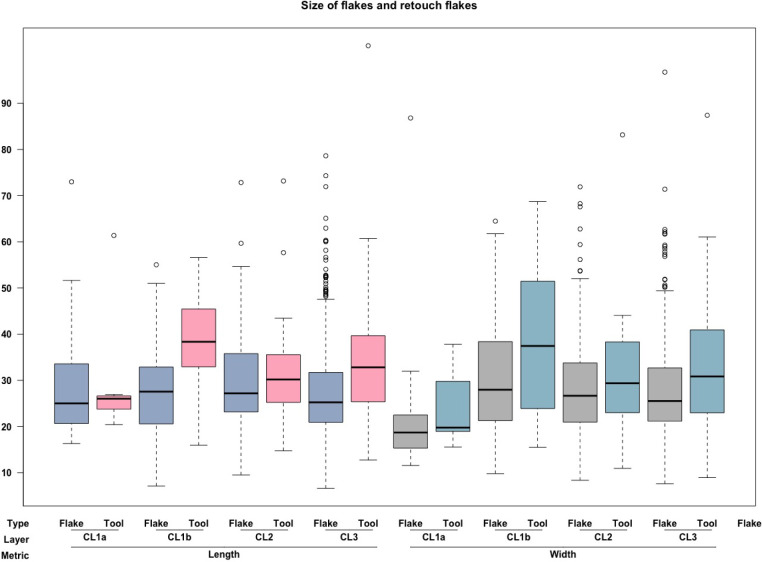
The length and width of flakes and tools.

We summarized the size of non-retouch flakes and retouched flakes of all the artifacts and the complete pieces (Tables 3 and 4). The two artifact groups show no notable difference. Based on complete blanks, flake length is nearly identical to its width (*Z* = -0.496, *p* = .62), which falls short of the conventional blade definition (technological length > 2*width). The length/width ratio obtained from complete flakes across most of the cultural layers also demonstrates this pattern, consistently being around a value of 1 across almost all layers at SDG2 for both non-retouched and retouched pieces. This trend is observed from CL3 to CL1b (Fig 4). The only exception is CL1a, which contains slightly longer flakes than the other layers. In other words, blank production across the CFAs shows a clear similarity in flake length and width, with little sign of narrow and elongated flakes. Additionally, retouched tools are significantly longer (*U* = 31423, *p* < .005) and wider (*U* = 37406, *p* < .005) than blanks but not thicker (*U* = -4.1, *p* = .029) throughout all assemblages. Except for the CL1a again (Fig 4), the difference in the size of retouched tools and blanks is not as clear. In summary, there appears to be a consistent blank production pattern at SDG2, aiming toward the production of relatively broad blanks and prominently larger sized tools.

**Table 3:** Measurements of blanks (mm).

		All flakes			Complete flakes			
		Length	Width	Thickness	Length	Width	Thickness	L/W
CL1a	Mean	28.68	19.19	9.085	29.15	22.05	9.68	1.48
	Median	24.50	17.65	8.59	25	18.69	8.8	1.46
	*SD*	10.8	9.57	3.93	13	14.9	4.58	-
CL1b	Mean	32	23.37	8.64	27.44	30.06	9.34	0.91
	Median	29.48	21.77	7.72	27.5	27.84	8.57	0.99
	*SD*	9.84	8.02	4.01	8.62	11.2	4.65	-
CL2	Mean	35.23	25.42	10.34	30.72	29.19	10.69	1.05
	Median	31.73	23.46	8.96	27.7	26.57	9.3	1.04
	*SD*	12.7	10.1	5.29	11.2	12.6	5.51	-
CL3	Mean	33.01	23.80	10.24	27.42	27.68	10.52	0.99
	Median	29.87	23.80	9.08	25.21	25.54	9.21	0.99
	*SD*	10.8	8.48	5.10	10.1	10.4	5.49	-
Total	Mean	32.94	23.77	9.85	27.99	28.20	10.29	0.99
	Median	29.96	22.03	8.63	25.89	25.93	9.09	1
	*SD*	10.96	8.74	4.91	10.14	11.21	5.33	-

**Table 4:** Measurements of tools (mm).

		All retouched flakes			Complete retouched flakes			
		Length	Width	Thickness	Length	Width	Thickness	L/W
CL1a	Mean	30.96	23.70	9.68	29.8	24.36	11.07	1.24
	Median	26.59	19.86	8.38	26.01	19.76	8.37	1.31
	*SD*	11.2	8.37	5.17	14.1	9.03	6.83	-
CL1b	Mean	37.57	28.99	9.1	38.24	38.72	10.57	1.1
	Median	34.64	27.7	8.15	38.34	37.44	9.85	1.02
	*SD*	12.5	11.3	3.87	12	18.2	5.12	-
CL2	Mean	37.55	28.59	10.47	32.62	30.34	9.24	1.2
	Median	32.72	28.27	9.04	30.18	29.37	9.04	1
	*SD*	15.4	12.7	7.17	11.6	14.3	5.22	-
CL3	Mean	38.28	29.03	12.68	33.90	32.67	12.62	1.04
	Median	35.63	26.91	12.68	32.06	30.87	11.04	1.04
	*SD*	12.8	11.6	6.69	12.6	13.4	7.22	-
Total	Mean	35.18	28.59	11.74	33.63	32.01	11.54	1.05
	Median	35.11	26.06	10.24	32.26	30.77	10.45	1.05
	*SD*	13.24	11.63	6.59	12.33	13.89	6.71	-

#### Platform and preparation

Here, we focused on the blanks and tools with platform preserved. In general, there is a lack of platform preparation in CFAs at SDG2. For flakes with platform preserved (N = 1013), the majority of the flakes and tools are characterized by plain platforms, followed by cortical, and with a small portion of crushed, punctiform, linear, dihedral, and facetted types (Table 5), as well as two other undetermined types. For blanks, the average platform thickness and width is 7.55 mm and 18.62 mm respectively, smaller than the platforms of retouched tools (average platform thickness = 8.29 mm; average platform width = 21.37 mm). This may be associated with the noted pattern that retouched artifacts tend to be larger than the unretouched flakes. The average exterior platform angle of retouched tools and unretouched flakes are 75.8° and 79° respectively. Based on flakes and tools with platform preserved, we observed instances of preparation on the exterior platform margin (PEPM), primarily battering and abrasion and two examples of facetted. We identified PEPM on 10.4% and 12.69% of the blanks and tools respectively: blanks mostly occur in CL2 and CL3; tools occur exclusively in CL2 and CL3 (Table 6).

**Table 5:** The frequency of platform.

	Plain	Cortical	Linear	Punctiform	Crushed	Dihedral	Facetted
CL1a	19 (50%)	7 (18.4%)	5 (13.2%)	5 (13.2%)	1 (2.6%)	1 (2.6%)	-
CL1b	119 (62.3%)	42 (22%)	9 (4.7%)	6 (3.1%)	6 (3.1%)	7 (3.7%)	1 (0.5%)
CL2	90 (56.3%)	26 (16.3%)	13 (8.1%)	5 (3.1%)	13 (8.1%)	4 (2.5%)	8 (5%)
CL3	389 (62.3%)	130 (20.8%)	28 (4.5%)	13 (2.1%)	29(4.6%)	12(1.9%)	23 (3.7%)
Total	617 (60.9%)	205 (20.2%)	55 (5.4%)	29 (2.9%)	49 (4.8%)	24 (2.4%)	32 (3.2%)

**Table 6:** The frequency of abrasion and battering on the exterior platform margin.

	Flakes with platform *N*	Frequency of PEPM *N(f)*	Tools with platform *N*	Frequency of PEPM *N(f)*
CL1a	30	1 (3.3%)	8	-
CL1b	182	6 (3.3%)	9	-
CL2	128	14 (10.9%)	32	11 (34.37%)
CL3	539	70 (13%)	85	6 (7.06%)
Total	879	91 (10.4%)	134	17 (12.69%)

#### Cortex and dorsal pattern

**Fig 5:**
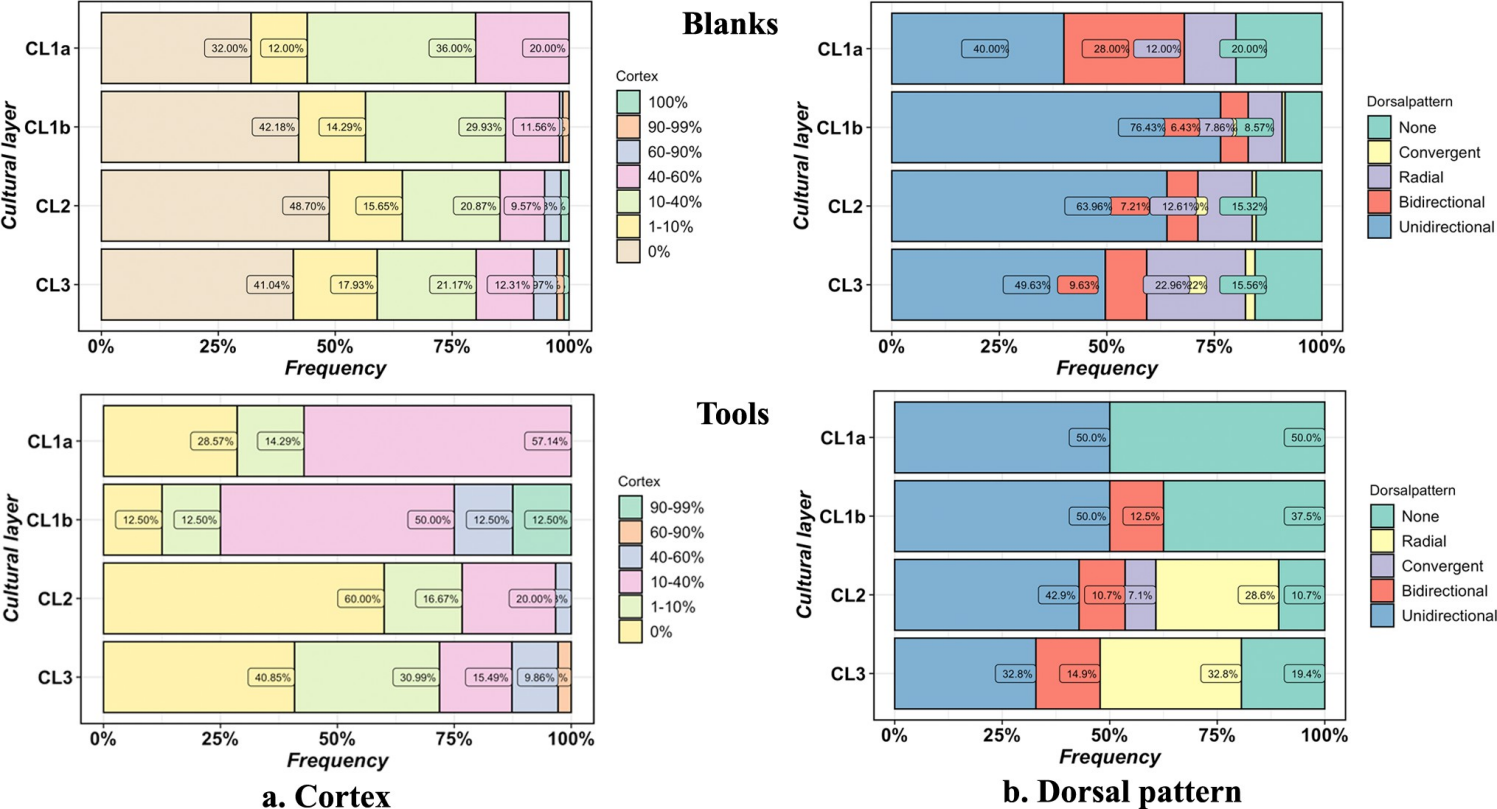
Cortex and dorsal pattern of flakes and tools.

More than half of the complete tools and blanks still contain some cortex (Fig 5A). For the blanks, about half contain either little or no cortex, and the rest is mostly with 10–40% and 40–60% of cortex. The proportion of flakes with slightly more cortex coverage increased in CL1a, about 70% the blanks include at least 1–10% cortex. Among the retouched tools, there is a relatively sharp increase in cortex from CL3 and CL2 to CL1a and CL1b. In CL2 and CL3, it is with around half with no cortex, and in contrast, the proportion of 10% and 10–40% cortex coverage increased notably in CL1b and CL1a. Dorsal scar pattern of flakes can be informative on flaking methods and reduction patterns.

Dorsal patterns on complete blanks (N = 681) and tools (N = 109) show that unidirectional is the most common pattern in both blanks and tools; followed by bidirectional, radial, and ‘no dorsal patterns’ (lacking identifiable dorsal scars, e.g., natural surface due to breakage) (Fig 5B). There are only a few examples of a convergent scar pattern in CL3 to CL1b. Across cultural layers, the frequency of the unidirectional scar pattern among blanks is the highest in CL2 and CL1b, while a relatively high rate of bidirectional patterns is present in CL1a. There are some differences in the dorsal pattern of retouched tools, although unidirectional pattern remains the major scar arrangement from CL2 to CL1a. In CL1a and CL1b, there are more tools without a clear dorsal pattern. Tools also show a slightly higher frequency of radial scars than in flakes from CL2 and CL3.

### Core reduction

**Fig 6:**
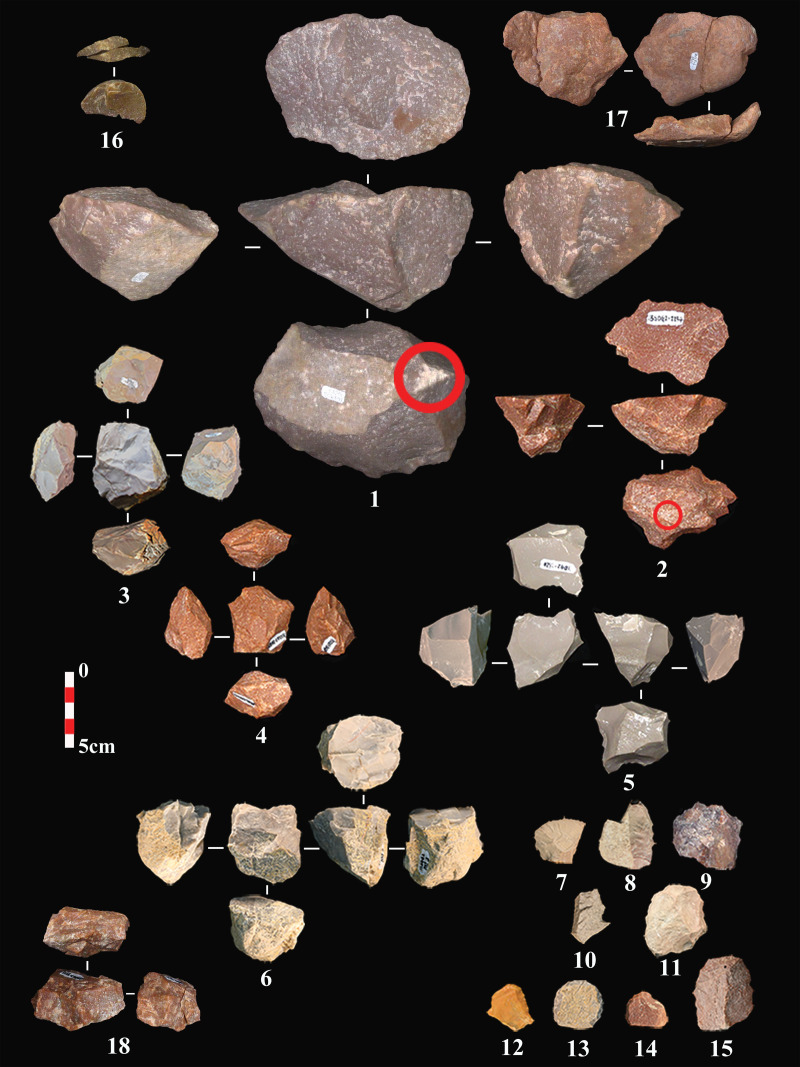
A few examples of cores and tools at SDG2. Single-platform cores (anvil-assisted cores?) (1–2); Discoid-like/alternating flaking cores (3–4); Multi-platform cores (5–6); Side scrapers (7–8); Denticulate (9); Notch (10); Splintered piece (11); End scrapers (12–15); Refits (16–18).

A total of 361 cores were uncovered from CL3 to CL1a. A significant number of cores (N = 119, 33%) shows only one or two flake scars and/or lack of clear platforms and appropriate flaking angles. The latter are considered here as tested cobbles/cores. CL1a and CL2 have higher frequencies of objects, with the former layer including more than half of the tested cores (Table 7). Most of the cores (and flakes) are consistent with the use of freehand percussion and hard hammer techniques. There is a lack of known elaborate reduction methods and instead, we observe short removal sequences and opportunistic core rotations (Fig 6: 5–6, 18). There are a few bipolar cores, core-on-flakes, and discoid-like/alternating-flaking cores (Fig 6: 3–4). Discoid-like cores are mostly derived from CL3 and CL2, while bipolar core frequency is slightly higher in CL2 to CL1a. As the cores were reduced with little to no help of preparations or standardized methods, we rely here on a simple classification system based on the number of striking platforms (Table 8). It shows that most layers have a relatively consistent number of the three platform categories and a higher number of single-platform cores, except for CL1b where multi-platform cores are more frequent. We also note two cores in CL3 and CL2 stand out by their pyramidal shape, turning reduction pattern, and pecking/percussion points at the distal end opposed to the flake platform (Fig 6: 1–2).

**Table 7:** Core types over layers.

	CL1a	CL1b	CL2	CL3	Total
Bipolar cores	3 (7.5%)	4 (11.1%)	9 (19.6%)	3 (1.3%)	19 (5.3%)
Core-on-flake	-	3 (8.3%)	1 (2.2%)	25 (10.5%)	29 (8%)
Discoid-like cores	1 (2.5%)	-	4 (8.7%)	7 (2.9%)	12 (3.3%)
Tested cobbles	26 (65%)	11 (30.6%)	18 (39.1%)	64 (26.8%)	119 (33%)
Other freehand cores	9(22.5%)	17 (47.2%)	12 (26%)	128 (53.6%)	168 (46.1%)
Undetermined	1 (2.5%)	1 (2.8%)	2 (4.3%)	12 (5%)	16 (4.4%)
Total	40 (100%)	36 (100%)	46 (100%)	239 (100%)	361 (100%)

**Table 8:** Core categories by platform over layers.

	CL1a	CL1b	CL2	CL3	Total
Single-platform cores	16 (40%)	8 (22.2%)	15 (32.6%)	100 (41.8%)	139 (38.5%)
Double-platform cores	14 (35%)	10 (27.8%)	10 (21.7%)	69 (28.9%)	103 (28.5%)
Multiple-platform cores	6 (15%)	13 (36.1%)	8 (17.4%)	53 (22.2%)	80 (22.2%)
Undetermined	4 (10%)	5 (13.9%)	13 (28.3%)	17 (7.1%)	39 (10.8%)
Total	40 (100%)	36 (100%)	46 (100%)	239 (100%)	361 (100%)

**Fig 7:**
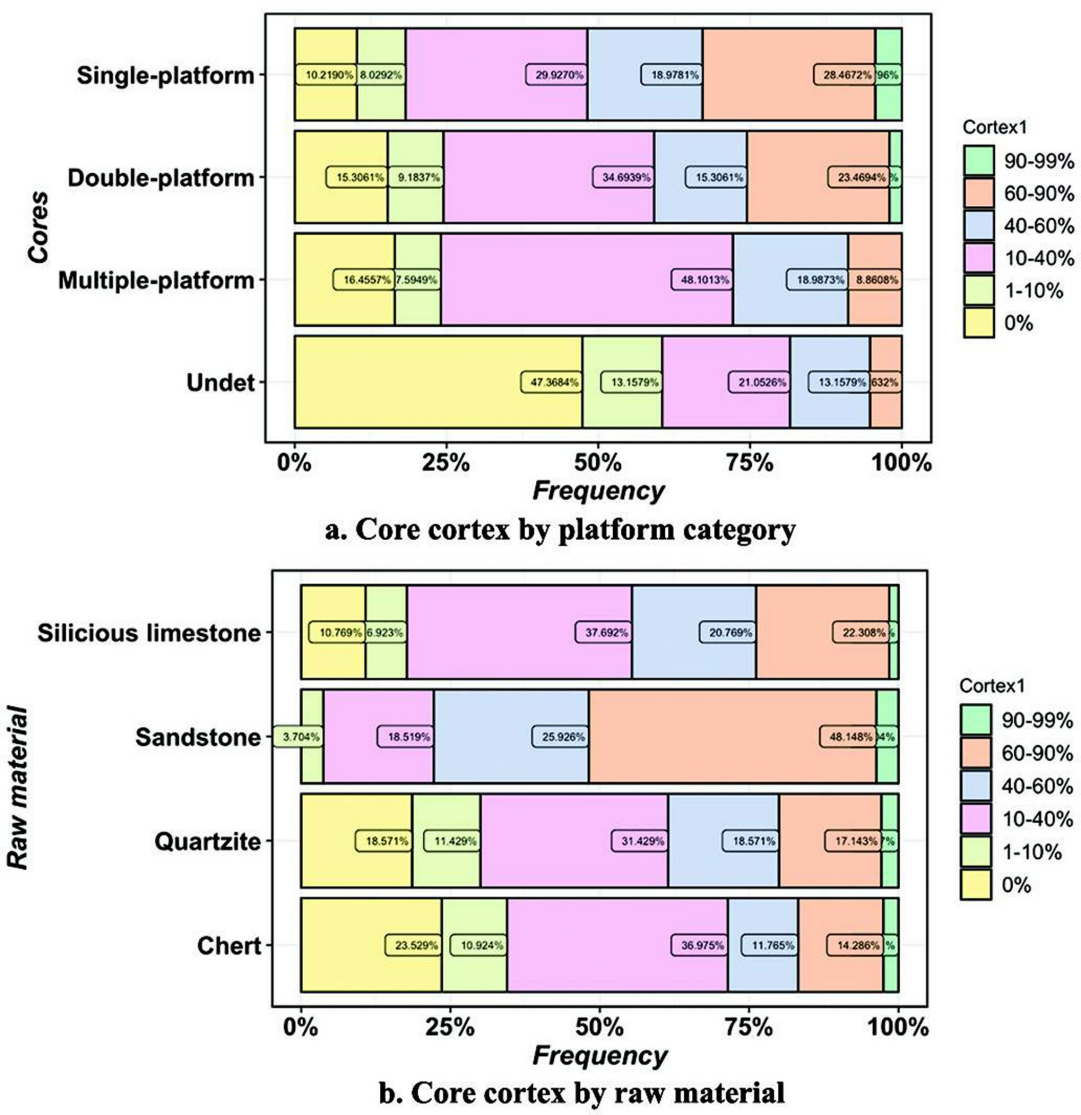
The core cortex by core platforms and raw material.

For the cortex on cores, we observe a decrease in cortex coverage from single-platform cores to multi-platform cores (Fig 7A). About 80% of the multi-platform cores have less than 40% cortex. It suggests that the number of platforms increases with reduction intensity. When raw material is taken into account (Fig 7B), sandstone cores has the highest cortex coverage and followed by silicious limestone, quartzite, and chert.

**Fig 8:**
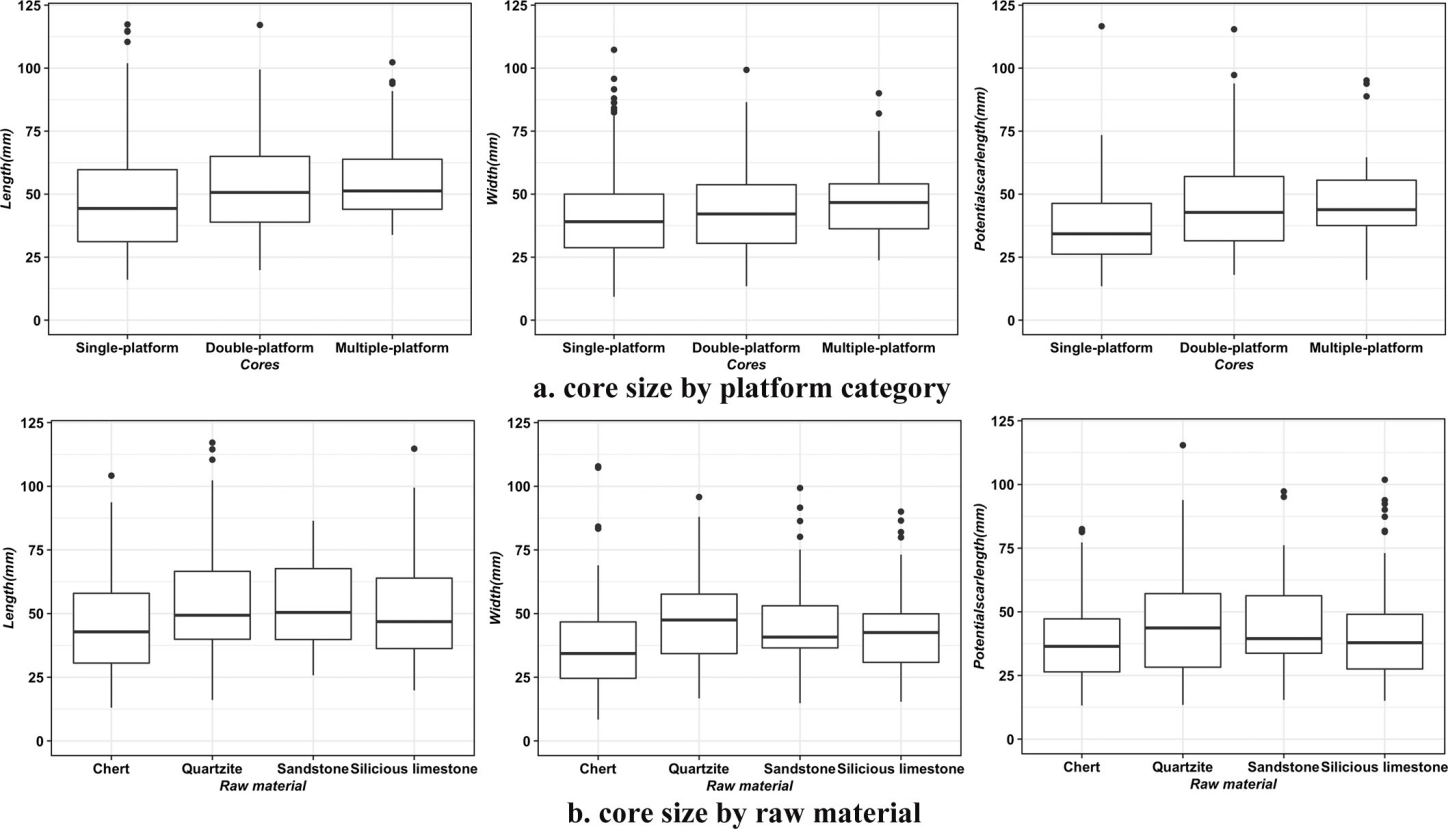
The comparison of core length, width, and potential scar length.

When we compared the length and width of the cores by the number of platforms (Fig 8), we observe a positive relationship between core size and the number of core platforms. It shows that cores with more platforms have larger sizes. To better understand the selection on the size of core cobbles at site, we compared the size of tested cores (as a proxy for original blank size) and the rest of cores (S1 Fig), which reports no significant difference. These findings suggest that, although the number of platform may increase as the core reduces, core size is a poor predictor for reduction intensity. Hence core blanks may vary in size, and larger cores were more intensively reduced.

Difference in core size between raw material types is observed (Fig 8B). Sandstone cores have more cortex and the biggest core size, whereas chert cores have the smallest sizes with the least cortex coverage. Quartzite and silicious limestone cores are in between, and the former cores are relatively bigger than the latter. To explore the core reduction further, we recorded an attribute called ‘potential scar length’, which is the maximum length that a blank could attain if the flaking surface on the cores was fully exploited. This measurement is meant to help us understand whether the maximum blank length is closer to the longest axis of cores or not. As shown in Fig 8, the potential scar length among cores is significantly different from core length (W = 76690, p < .005) and are instead much closer in line with core width (W = 63590, *p* = .42). This indicates that the cores might be generally reduced along the secondary axis rather than the longest axis of the core; the latter would have been the optimal to obtain elongated flakes or blades. All in all, the cores among CL3-CL1a show no evidence of standardized forms for targeting the production of blades, nor reduction removal patterns associated with laminar products.

### Tool modification and raw material exploitation

**Fig 9:**
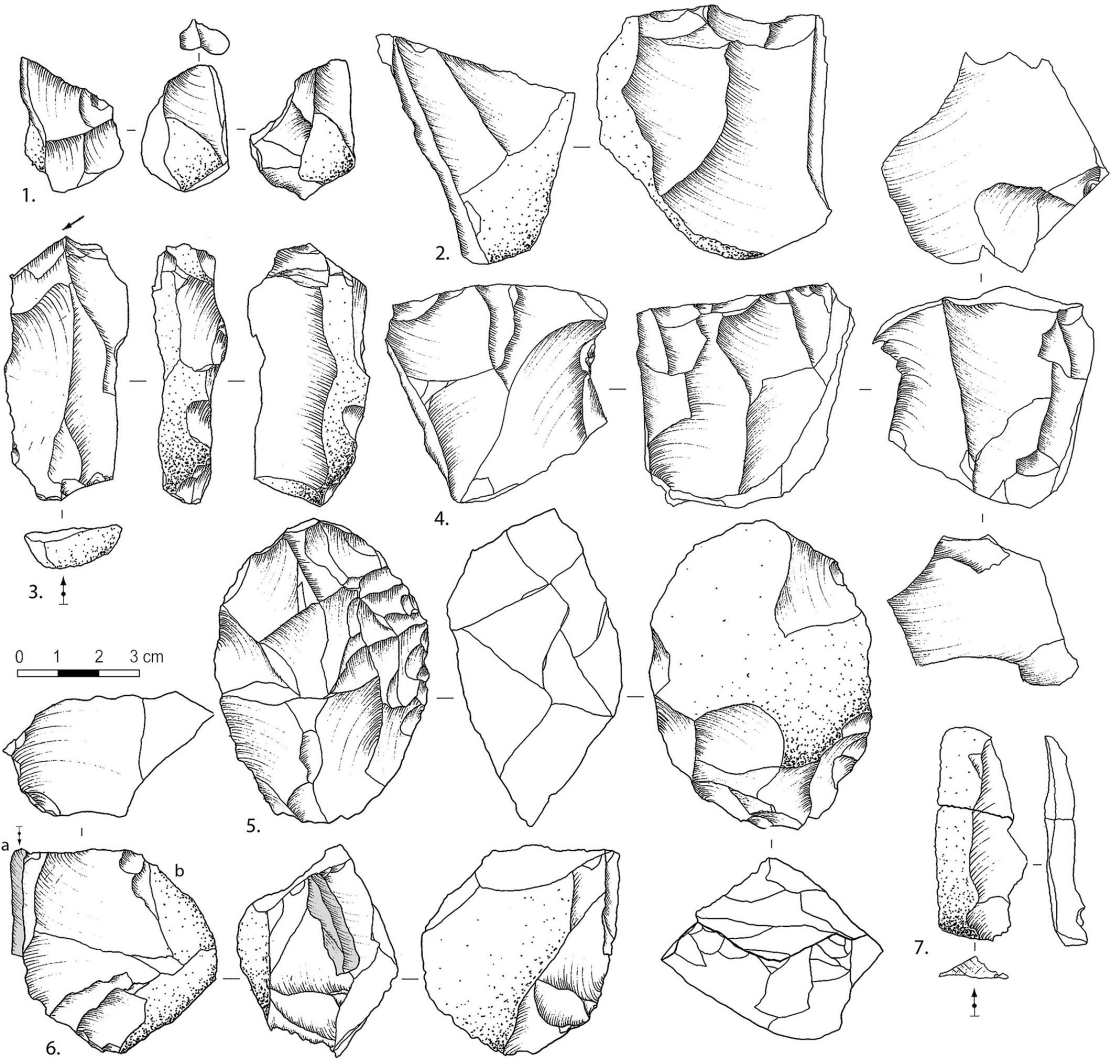
Variations in core shape and reduction method at CL3. A few known examples of atypical blank forms and core types in low frequency. 1, 2; 4–6, cores; 3, 7, flakes. Small flake core (1); Unidirectional asymmetrical core (2); Pseudo-burin on core-edge transverse flake (3); Bidirectional core (4); Discoid-like core (5); Discoid-like core (b) with a refitted accidental bladelet removal (a) (6); Elongated flake (7) (drawings by N. Zwyns).

Retouch tools were made from flakes (N = 217, 73.4%), shatter (N = 58, 19.9%), and cores (N = 17,5.8%). In terms of typology, the set is dominated by sidescrapers (N = 168, 57.5%), and other tool types such as are notches (N = 40), denticulates (N = 18), end scrapers (N = 13), splintered pieces (N = 8), points (N = 8), perforators (N = 3), and some unclassified retouched pieces (Fig 8: 1–4, 6–8; Fig 9: 2, 3, 5, 6, 10–12, 15–17). Some flakes without clear modification were shown to be utilized as tools through use-wear analysis of their edges [65]. Given this, the frequency of retouched tools may not provide an accurate estimation of tool frequency or blank utilization [66]. All denticulates were found in CL3, but other tool types have not varied by layer. Retouch localization and delineation does not vary significantly either: direct retouch (N = 162, 55.5%), followed by alternate (N = 64, 21.9%) and inverse (N = 43, 14.7%), and a few bifacial; while the edges are mostly straight (N = 116, 39.7%), convex (N = 53, 18.2%) or irregular (N = 51, 17.46%), with some concave, denticulate.

**Fig 10:**
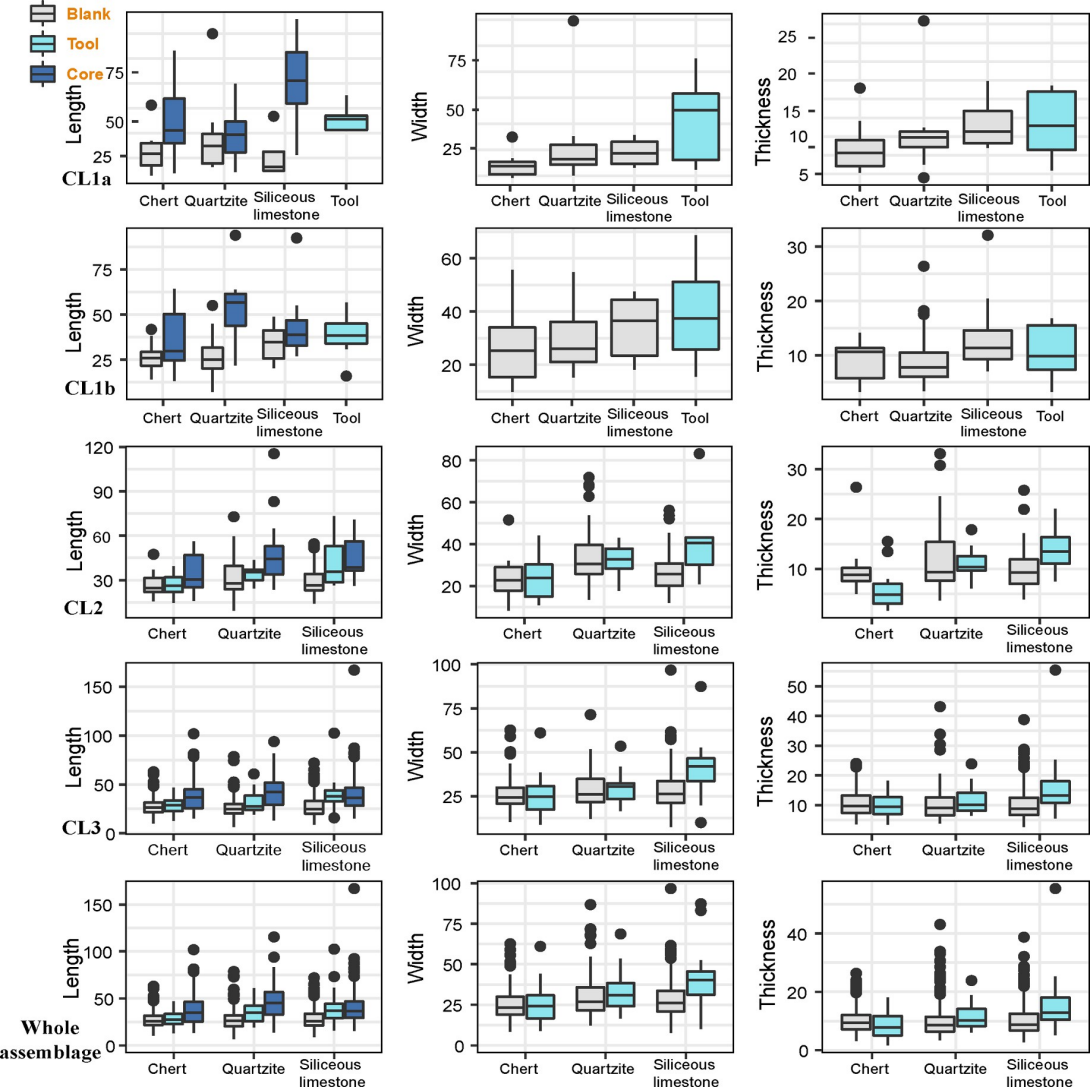
Size of flakes and tools of three raw materials. The length, width, and thickness of blanks (grey), tools (light blue), and cores (dark blue); In CL1b and CL1b, due to small sample size, all tools were grouped together regardless of raw material.

As suggested earlier, the size of the tools is significantly larger than the blanks at site (Fig 4). We further investigated whether the pattern relates to raw material exploitation or not. We compared the size of complete blanks and tools made on the three major raw material types: chert, quartzite, and siliceous limestone, for CL2, CL3, and the whole studied sample (Fig 10). Due to limited sample size of complete tools in CL1b (N = 8) and CL1a (N = 7), the tools from the two layers were grouped together regardless of raw material (Fig 10). The overall trend of tools being larger than flakes appears to be driven mainly by siliceous limestone tools being consistently larger than the flakes of the same raw material in length (Z = 8085.5, p < .005), width (Z = 8313.5, p < .005), and thickness (Z = 7808, p < .005), especially in CL2 and CL3. In comparison, there is no clear difference between tool and blank size among chert and quartzite artifacts. Additionally, there is a greater degree of overlap in the length of cores and tools for siliceous limestone than the other two materials. These results indicate that those larger blanks of siliceous limestone were preferentially selected for further tool modifications, perhaps because the relatively fine-grained nature of siliceous limestone may facilitate more predictive fracture propagation, allowing knappers to have better a control in producing larger flakes.

## Discussion

### The diachronic variation of the CFAs

#### Shared technological features

**Fig 11:**
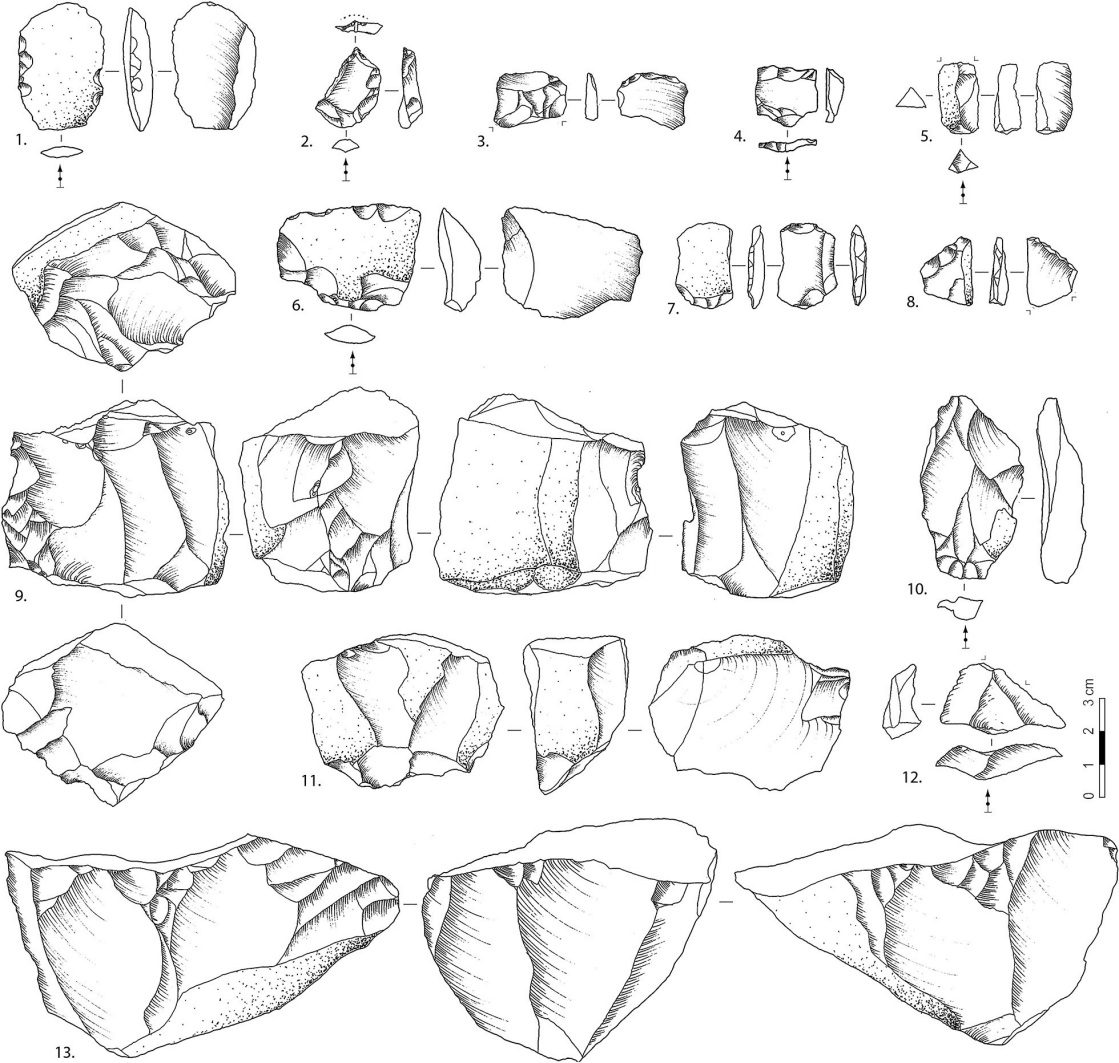
The illustration of variability in technological markers at CL1b. A few technological variations accrued low frequency at site. 1–4, 6–8: retouched/used flakes; 5, 10, 12: flakes; 9, 11, 13: cores. Retouched cortical flake (1); Perforator (2); Flakes with used edge (?) (3, 4, 8, 7); Multiple platform core (9); Levallois Flake (10); Type 1—Core on flake (11); Pseudo-Levallois point (12); Anvil-assisted core (?) (13) (drawings by N. Zwyns).

Overall, the lithic productions represented in CL3-CL1a is characterized by low investment in core preparation and expedient reduction. Few cases of predetermination can be inferred from the observed reduction sequence, and the retouched tools lack standardization. The raw materials selected were mostly local siliceous limestone, chert, quartzite, and sandstone from the Biangou riverbed. Cores were generally reduced by direct percussion, probably with hard hammers. The tested cores and core cortex indicate that many cores were abandoned quickly, without intense reductions, and some other cores might have been transported elsewhere [67]. Chert, quartzite, and silicious limestone cores are more reduced than sandstone ones, by prolonged single-platform reduction or by simple rotations leading to double- and multi- platform cores (Fig 6: 1–2; 5–6; Fig 9: 1, 2; 4–6). Although multi-platform cores have slightly heavier reduction based on cortex proportions (Figs 7 and 9: 2, 4–6; Fig 11: 9), they have larger core sizes than other cores (Fig 8). There are two possibilities for this: a higher number of platforms and flaking surfaces do not necessarily suggest a more advanced reduced stage among the single- or double-platform cores; or larger cobbles were selected for heavier reductions.

The lack of preparations among the CFAs aligns with the general expedient characteristic of lithic production, indicating that very limited efforts were made in predetermination. The majority of the flakes are produced by basic flaking removals, an observation consistent with the short sequences of ‘salami slicing’ or unidirectional flaking as suggested from technical refits (Fig 6: 16–18). Together with the examination on blanks, there is no clear effort in the production of elongated flakes after 34 ka cal BP at SDG2. Retouch shows neither standardization in shape nor curation. Yet retouched tools are larger than blanks, specifically among the siliceous limestone tools, indicating selection on the size of blanks for tool modification. The abundant raw material availability at SDG2 might be one of the reasons for this low-cost reduction strategy which characterizes the lithic production from 34 ka cal BP to 28 ka cal BP.

#### Temporal variations

That are signs of temporal changes in flaking methods and techniques from CL3 to CL1a. For example, the frequency of abrasion and battering on the exterior platform surface increased from CL3 to CL2 before declining sharply in CL1b and CL1a. The frequency of tool retouch also decreased in CL1b and CL1a. In terms of core reduction, flaking patterns involving the alternate use of two flaking surfaces as platforms occurred in CL3 and CL2 (Fig 6: 3–4; Fig 9: 5–6) but not in CL1a and CL1b. Some were reduced by alternating flaking on two nondistinctive surfaces (Fig 6: 6). A few examples show cortex preserved on one face (Fig 9: 5), which may indicate hierarchical conception of the reduction process and removals parallel to the intersection plane separating the two flaking surfaces. The reduction method associated with these few cores is relatively close to discoid core reduction in its broadest sense [68–70] with signs of peripheral convexity and removals secant relative to the intersection plane. However, there are no convincing signs of pre-determination on cores; hence it is difficult to compare with those typical unifacial discoid reduction [71, 72] or centripetal Levallois [69]. Discoid knapping method is found in the western Loess Plateau since at least the MIS6 and other regions of China, which is not temporally and regionally constrained as a special technological feature during the Paleolithic period [73].

Another example of derived technical trait in CL2 and CL1b relates to the pecking/grinding traces observed on the surface of some pyramidal-shape cores (Fig 6: 1–2). It might be possible that these impact traces were associated with the use of these cores as hammers, retouchers or pecking tools. Alternatively, given that these traces tend to occur on prominent edges located at the distal end of flaking surfaces, it is also possible that these cores were knapped with a ‘anvil-assisted/anvil-rested’ approach [74, 75]. That is, the cone-shaped core with a flat plain surface used as the platform was placed on an anvil and flaked by turning or semi-turning removals. This technique differs from axial bipolar flaking [76], which is initialized by the split/compression force of the anvil counterstrike to break core under an angle about 90° [77], at the expense of control over the flaking process. With the ‘anvil-assisted’ technique identified here, the anvil would be merely providing a surface to stabilize/rest the core rather than using the rebound necessary for axial bipolar flaking [78]. This flaking method may increase the number of flakes obtained per core [74], and we note that CL1b indeed shows the highest flake frequency.

Other examples of derived, yet unusual, features include cores-on-flakes (Fig 11: 11), which indicate occasional branching of reduction sequences through ramification [79]. From a typological point of view, we note that only a few flakes may overlap with the definition of the Levallois flake ‘type’ (Fig 11: 10), but there are no other signs of Levallois reduction in the assemblages. The same goes for the few artifacts that fulfill the typological criteria of a pseudo-Levallois point, but there is no additional clear evidence for discoid reduction either (Fig 11: 12). These observations warn against the use of isolated artifact type as markers for specific flaking methods since these cases may reflect stochastic variation from accidental productions rather than designed systems.

In addition, we highlight three trends observed across the cultural layers. First, the flake frequency increases gradually from CL3 through CL1b (Fig 3). This observation is supported by the ratio of flake/core and tool/flake across the cultural layers (based on blanks and tools with platform preserved; Table 9). It shows a general trend of increasing flake productivity through time, especially the notably high flake productivity in CL1b. In CL1a, the low flake/core could be related to the high frequency of tested cores at this layer. We also observed a slight increase in the use of quartzite over time, especially in CL1a and CL1b, a material that is coarser and harder to control for flaking than siliceous limestones and cherts. As the frequency of quartzite retouched tools increased, the use of chert decreased, while both show a low rate of retouch/tool modifications. The presence of artifacts made on non-local chert (especially tools or flakes with use-wear identified) in CL2 has been suggested to reflect increased transport distance and mobility during this phase [63, 65]. These features seem to indicate that there were many changes in CL1b and CL1a from lower layers during the period of the transition from MIS3 to MIS2. However, previous studies on cortex ratio indicates that assemblage cortex is consistently underrepresented among all four upper cultural layers and across all major raw material types, meaning a persistence in artifact transport away from SDG2 in the late MIS3 [64, 67].

**Table 9:** Ratios of flakes per core and tools per flake.

Ratios	CL3	CL2	CL1b	CL1a
Flake/core	2.75	3.64	5.3	0.95
Tool/blank	0.14	0.2	0.05	0.21

To summarize, our analyses do not show significant changes in the overall technologies and techniques used for blank production, core reduction, or tool modification over layers that postdate 34 ka cal BP. Although it seems that there was more effort in the retouch of tools and platform preparation in CL3 and CL2, as well as a slightly higher use of the raw material of siliceous limestone and chert. The lithic production strategy at the site generally illustrates a similarly expedient manner. The assemblages of the upper layers are unambiguously flake-based without signs of elongated flakes and little evidence for standardized tool retouch, but with a selection of the large flakes for retouch (particularly with silicious limestone artifacts). Except for a very small number of exhausted tools made of exotic materials from CL2, few exhausted cores/tools in the assemblages overall perhaps reflect the proximity to raw material source. The general raw material abundance could partly explain the fact that there are low selective pressures on the production of predetermined blanks and formal tools [80]. There are, however, minor variations such as the occurrence of alternate flaking methods (CL3-CL2), or the occasional use of anvil-assisted technique or cores as hammer stones (CL2-CL1b). Given their overall low frequencies throughout the cultural layers, it is currently difficult to relate these features to broader technological patterns at SDG2. It is notable, however, that unambiguous blade technology is not present in the CFAs of these upper layers.

### After the blades

#### The technological shift back to CFAs in a changing environment

Considering the bulk of the chronological data available for SDG1 and SDG2, earlier dates are more reliable than the few late ones and place the blade assemblages between 41 and 34 ka cal BP [9, 28, 29, 31, 32, 81]. These dates are broadly consistent with a scenario of human population dispersals from the Eurasian Steppe [20, 21, 25, 26], from North Mongolia [27, 82], and/or the Altai [53]. After 34 ka cal BP, however, the unequivocal predominance of CFAs in North China suggests that the occurrence of blades is only a short episode in the region’s technological sequence. Our analysis confirms that the CFAs postdating the IUP artifacts do not contain any reminiscence of the laminar technology, nor do they show derived bladelet productions that are common in the EUP to the north and to the west. Although elongated flakes were occasionally observed, they are not the result of a specific effort of blade production, but rather the products of straightforward reduction techniques. For example, one of the several bladelets found in CL3 (Figs 11 and 6A) was refitted to an alternating flaking core (Fig 10: 6b), suggesting that the removal was accidental instead of from predetermined knapping sequences. In short, our results are in line with characterizations of the SDG2 CFA productions as expedient [65].

The technological evolution documented at SDG2 is a clear and unusual technological reversal from the blades back to a non-predetermined, low-cost approach of flake production that was locally widespread during the late MIS3. This reversal is in contrast with what is observed in the Eurasian Steppe at that time (Fig 1), where the IUP is generally followed by an early Upper Paleolithic defined by the emergence of bladelet production systems [23, 37]. Contrary to the Steppe where the laminar traditions became consistent components in regional technological evolution, a different technological trajectory was present in the adjacent region of North China. After its sudden appearance around 41 ka cal BP, the blade technology disappeared by 34 ka cal BP at SDG2, leaving no identifiable remnant in the studied lithic assemblages. The lack of blade elements in these later assemblages cannot be explained solely by deposition processes, because no significant post-depositional transport has been identified [30, 55]. Nor can it be easily attributed to raw material quality or availability as the blade assemblages from SDG1 and SDG2 are made on the same local raw materials as the CFAs [53, 54]. SDG2 was described as a residential camp [50, 63], with a lithic production characterized by an expedient pattern and showing a low frequency of artifact recycling.

As mentioned above, the timing of this technological shift during the late MIS3 was marked by frequent millennial-scale climatic oscillations [39, 40]. Located on the western edge of the Loess Plateau, SDG sits at the boundary between the East Asian summer monsoon and winter monsoon regions [83, 84], and relatively close to the intersection between the Palearctic and Oriental biogeographic landscapes [85–87]. The biogeographic interface separating the continental landscapes and the relatively humid summer monsoon regions would have shifted latitudinally (advancing and recession) as the climate fluctuated during the late MIS3 [88]. At SDG2, the technological changes observed are associated with a shift in sedimentary regime from a lacustrine to terrestrial [30, 32]. Furthermore, we note that the CFAs in North China tend to cluster in the areas within the East Asian summer monsoon regime (Fig 1), whereas IUP assemblages are mostly found in the north with inland continental climates. Overall, the distribution of technologies is consistent with the biogeographical extension of the East Asian monsoon. Hence, the fluctuating boundaries between eco-zones may partly explain the distribution of the two technological systems during the MIS3. For example, with a weakening East Asian summer monsoon and a retreat of the monsoon range southward, the climate around SDG could have become drier and colder, which in turn triggered a change in hunter-gatherer mobility and associated technological shift, such as described in southern Africa [89]. The expansion of the steppe may have facilitated a concomitant expansion of IUP blade technology and associated subsistence strategies into North China. On the contrary, the advancing of summer monsoon might have brought the flake-based production back in the region. However, correlation is not causality and testing this hypothesis would not only require a finer resolution in environmental reconstructions, but also a more accurate description of the adaptive mechanisms between human behavior and environmental pressures involved. Along with the impact of environment, factors of mobility pattern, population movements, and demographic history could also help explain the technological changes observed at SDG2.

#### Mobility patterns and/or population dynamics

Facing the environmental pressures described above, mobile foragers could have adapted their settlement patterns and provisioning strategies [18, 56, 90]. Under low environmental stress, the expedient character of the lithic assemblages close to a raw material source is commonly associated with residential base camps [91, 92]. The general idea is that hunter-gatherer groups settle on raw material sources, thereby experiencing little pressure to extend the utility and maintenance of their toolkits, or to rely on formal tools. Such situation would also be observed at the nearby site of Dadiwan, where reliable local quartz sources decreased the costs associated with the risk of failure in tool productions [56, 93, 94]. Although it is unlikely to account for the dominance of CFAs in a long term, at the larger scale of East Asia, shifts in settlement patterns might be among the behavioral adaptions involved in the resurgence of CFA technologies around 34 cal BP at SDG2.

The shift back to the very basic technology may be also linked with a general decrease in population size and/or density, in the context of a fragmented cultural landscape [95]. As opposed to larger population sizes promoting cultural innovations and their transmission locally, smaller population density could lead to a loss of cultural diversity [96–98]. Lycett and Norton [10] argued that the lack of “Mode 2” technology among CFAs in East Asia may be related to local population structure and cultural transmission. The impact of this ‘cultural drift’ would grow stronger as the population size decreases and cause a lack of innovations and the loss of specific technological traits within the region or population locally [99]. This demographic model has been applied to various archaeological settings involving transitions from so-called ‘complex’ to ‘simple’ technologies (see summary for examples from southern Africa [100]). At SDG2, however, ostrich eggshell beads, perforated freshwater mollusks, and the pendant found with CFAs in CL2 and CL3 are evidence of, derived Upper Paleolithic features. The development of such behavioral traits does not fit the prediction of a demographic collapse scenario in a strict sense [58, 59]. We would expect such a crash to affect the transmission of complex symbolic forms at least as much as it impacted the lithic technology, but instead, innovations such as ornaments flourish during that period. Keeping in mind that we know very little about the actual population size of these groups either, ornament such as OES beads are known both in the Steppe zone and southward, and could represent evidence of contacts between neighboring regions.

Others have suggested that the general persistence of CFAs in East Asia instead indicate a local population continuity [2, 11, 12], resistant to external influences. From this perspective, the appearance and disappearance of the blade assemblages at SDG may reflect discontinuous cultural contacts between local populations and those of the neighboring regions. The CFAs nonetheless lack of specific and intricate derived features, making it a weak proxy for drawing phylogenetic inferences and/or cultural inferences regarding past population structure [82, 101]. Moreover, the increasing evidence of personal ornaments along with the CFAs thereby suggests that, although the lithic technology has been generally persistent, there are other aspects of technological innovations occurring at a different pace in the region [58, 59, 102, 103].

Additionally, ancient DNA and fossil remains from Bacho Kiro Cave (Bulgaria) [46] and the contemporaneous Ust’-Ishim femur in West Siberia [45] reveal that the makers of the IUP are most probable *H*. *sapiens*. Moving south, a distinct *H*. *sapiens* lineage represented by the fossil individual from Tianyuan Cave rose in North China around 40 ka cal BP [41, 104]. Between 40 ka and 30 ka, this population extended to a large area covering from North China, the Amur (Heilongjiang) region, and to East Mongolia [42, 43], at the time of the technological ‘reversal’ to the CFAs. Furthermore, the Salkhit human skullcap (Dornod Aymag, Mongolia) shows contributions from Tianyuan-related ancestry (~75%) and from another population identified at Yana RHS near the Artic Circle (~25%) [43, 44, 105, 106]. The Tianyuan-related ancestry remains relatively isolated in the Amur region until another population expanded around the Last Glacial Maximum [43]. Both Bacho-Kiro and Tianyuan lineages contributed to ancestry of present-day East Asians, yet they are not directly related [41, 47]. Accordingly, existing evidence points to complex population dynamics and cultural interactions between North China and the Steppe belt during the MIS3, involving distinct human lineages, population groups, and technologies. In this context, technological shifts might reflect a singular aspect of broader, intricate processes. We acknowledge that linking lithic variations to human population history remains a notoriously difficult task that needs to be treated cautiously. Yet given the chronological and geographical overlap between genetic and archaeological evidence, population movements and interactions remain a plausible explanation for the technological shift at SDG2. Hence, it is worth to further study the technological evolution and population dynamics in the region.

To summarize, the technological shift observed at SDG2 is unlikely due to site formation processes, raw material availability, site function, or recycling of the material. Although the lithic assemblages lack derived features, other behavioral innovations such as personal ornaments are inconsistent with the hypothesis of demographic collapse. Instead, several scenarios involving population dynamics and/or an adaptation to environmental changes are more parsimonious. The observed technological shift illustrates a relatively sudden scenario rather than a gradual adaptive process, but it may still reflect drastic changes in human behavior related to population movements and/or under strong (if any) selective pressures. Recent genetic evidence supports a complex scenario of cultural interactions, population dynamics, and gene flow in North China and the neighboring Steppe in late MIS3. In fact, the frequent climate fluctuations leading to the expansion/recession of East Asian Monsoon may have turned the interface between two biogeographic regions into opportunities for social interactions and population movement. The resurgence and persistence of CFAs may reflect either or both situations.

## Conclusions

Our results indicate that the CFAs of SDG2 show minor variations between the cultural layers and subtle temporal trends affecting the raw material economy (e.g., increases in flake production or in the use of quartzite). The CFAs at SDG2 overall have more in common than difference in terms of lithic production, and the variations observed do not include any evidence of blade technology. The laminar production system that existed at the site between 41–34 ka cal BP disappeared without leaving technological relics in the regional record. Instead, the post-34 ka CFAs exhibit poorly standardized systems of tool manufacture and modification, as well as low investment in controlling the shape and size of the flakes produced. In short, in contrast to what is observed in the Eurasian Steppe at the same time, the assemblages of SDG2 presents a clear case of technological reversal back to the very basic and unprepared CFA production during the late MIS3 from the preceding blade technology.

We find no evidence that raw material availability, sampling bias, the visibility of technological elements, or the cultural loss from demographic collapse played significant roles in this unusual technological shift. Instead, it is more likely attributed to the adaptive response to environmental variations and/or population movements in the region. The reversal back to the CFAs at SDG2 are contemporaneous (geographically and chronologically) with the evidence for the scenario of cultural interactions, population movements and expansions, and gene flow between North China and the neighboring Steppe. Pending new analyses, the resurgence and persistence of CFAs may reflect either/or both situations. Acknowledging current data limitations, we expect that finer-resolution reconstructions for the chronology and the environment in the region will help testing these explanations, and further clarify what characterizes the late MIS3 population dynamics and lithic technological developments in and around North China.

## Supporting information

S1 Table Basic data of stone artifact of SDG2. (XLSX)

S2 Table The raw material of stone artifacts over layers. (XLSX)

S1 Fig Tested cores size. (TIF)
