# Supplementary figures and images for: After the blades: The late MIS3 flake-based technology at Shuidonggou Locality 2, North China
Source: PLoS One. 2022 Oct 12;17(10):e0274777. doi: 10.1371/journal.pone.0274777 (PMC9555678; doi:10.1371/journal.pone.0274777)

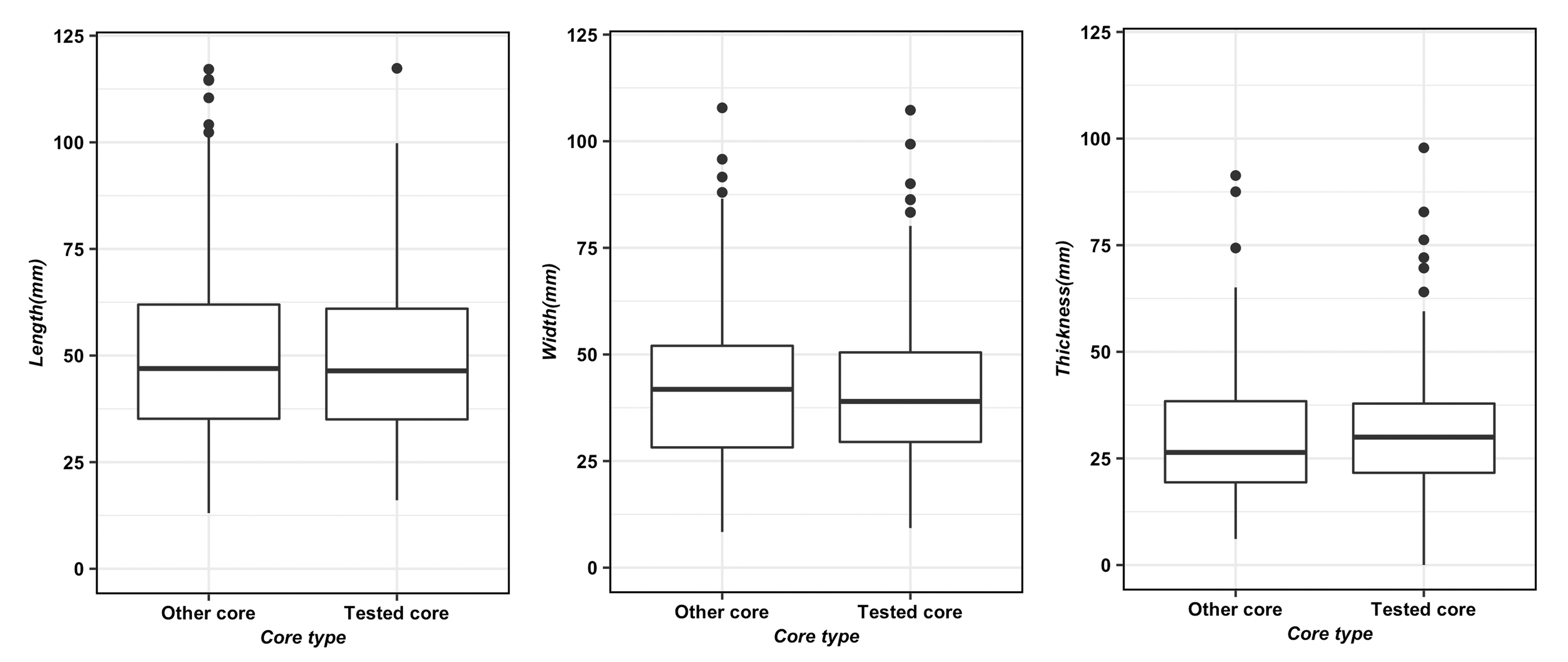

Supplement: S1 Fig — (TIF) [file pone.0274777.s003.tif]
